# The Bdnf and Npas4 genes are targets of HDAC3-mediated transcriptional repression

**DOI:** 10.1186/s12868-019-0546-0

**Published:** 2019-12-28

**Authors:** Anto Sam Crosslee Louis Sam Titus, Dharmendra Sharma, Min Soo Kim, Santosh R. D’Mello

**Affiliations:** 1 Department of Biological Sciences, Southern Methodist University, Dallas, TX USA; 2 Quantitative Biomedical Research Center, University of Texas Southwestern Medical Center, Dallas, TX USA; 3 Dallas, TX 75243 USA; 4 Present Address: Department of Molecular and Cell Biology, Baylor College of Medicine, Houston, TX USA; 5 Present Address: Department of Biomedical Engineering, University of Houston, Houston, TX USA

**Keywords:** HDAC3, Npas4, Bdnf, ChIP-Seq, Neurotoxic, Neurodegeneration

## Abstract

**Background:**

Histone deacetylase-3 (HDAC3) promotes neurodegeneration in various cell culture and in vivo models of neurodegeneration but the mechanism by which HDAC3 exerts neurotoxicity is not known. HDAC3 is known to be a transcriptional co-repressor. The goal of this study was to identify transcriptional targets of HDAC3 in an attempt to understand how it promotes neurodegeneration.

**Results:**

We used chromatin immunoprecipitation analysis coupled with deep sequencing (ChIP-Seq) to identify potential targets of HDAC3 in cerebellar granule neurons. One of the genes identified was the activity-dependent and neuroprotective transcription factor, Neuronal PAS Domain Protein 4 (Npas4). We confirmed using ChIP that in healthy neurons HDAC3 associates weakly with the Npas4 promoter, however, this association is robustly increased in neurons primed to die. We find that HDAC3 also associates differentially with the brain-derived neurotrophic factor (Bdnf) gene promoter, with higher association in dying neurons. In contrast, association of HDAC3 with the promoters of other neuroprotective genes, including those encoding c-Fos, FoxP1 and Stat3, was barely detectable in both healthy and dying neurons. Overexpression of HDAC3 leads to a suppression of Npas4 and Bdnf expression in cortical neurons and treatment with RGFP966, a chemical inhibitor of HDAC3, resulted in upregulation of their expression. Expression of HDAC3 also repressed Npas4 and Bdnf promoter activity.

**Conclusion:**

Our results suggest that Bdnf and Npas4 are transcriptional targets of Hdac3-mediated repression. HDAC3 inhibitors have been shown to protect against behavioral deficits and neuronal loss in mouse models of neurodegeneration and it is possible that these inhibitors work by upregulating neuroprotective genes like Bdnf and Npas4.

## Background

Histone deacetylases (HDACs) are enzymes that deacetylate histones as well as a large number of other proteins in the nucleus, cytoplasm and mitochondria. The 18 HDACs expressed in mammals are divided into two subgroups based on sequence similarity and activation mechanism—classical HDACs (HDACs 1–11) and Sirtuins (Sirt1–7) [1–3]. Chemical inhibitors of classical HDACs protect against neurodegeneration in a variety of invertebrate and vertebrate models of neurodegenerative diseases, suggesting that aberrant activation of HDACs promote neuronal death, however, studies conducted on individual members of the HDAC family suggests a neuroprotective effect for some of them [4–7]. For example, HDAC4, HDAC6, HDAC7 and HDRP (a truncated form of HDAC9) protect neurons from death [4, 8–12]. Recently, we identified HDAC3 as a protein with high neurotoxic activity and found that it promotes neuronal death in cell culture models including a Huntington’s disease (HD) model in which mutant huntingtin is overexpressed [13–15]. This neurotoxic action of HDAC3 involves its phosphorylation by glycogen synthase kinase 3β (GSK3β) and interaction with HDAC1 [13]. Consistent with the requirement of HDAC3 in neurodegeneration is the finding of protection by HDAC1/HDAC3-selective inhibitors against neuropathology and behavioral deficits in mouse models of HD and Fredereich’s ataxia [7, 16, 17]. HDAC3 orthologs have also been found to promote poly-Q toxicity in *Drosophila* and *C. Elegans* models of HD [18, 19]. Recent studies have described that HDAC3 protects against optic nerve injury-induced retinal ganglion cell death and combines with LRRK2 to promote neuronal death in a PD model [20, 21]. Another group has described pharmacological inhibition of HDAC3 restores amyloid-β induced impairment of plasticity [22].

While it is well accepted that HDAC3 has neurotoxic effects, how this is mediated is not known. It is known that Hdac3 represses gene transcription as part of the NCoR1/SMRT co-repressor complex [1–3]. It is therefore possible that HDAC3 promotes neurodegeneration by repressing the expression of genes that are required for neuronal survival or those genes that are stimulated in response to neurotoxic stimuli thereby protecting against them. While many targets of HDAC3 have been identified in non-neuronal tissue and cell types such as liver, macrophage and T cells [23–26], the genes regulated by HDAC3 in neurons or in the brain, particularly in the context of neurodegeneration, remain to be identified. To address this issue, we used ChIP-Seq to determine genome-wide binding of HDAC3 in healthy neurons and neurons primed to die. Among several others, our analysis identified the transcription factor, Neuronal PAS domain protein 4 (Npas4) and brain-derived neurotrophic factor (Bdnf), as potential targets for the HDAC3. Npas4 is an immediate early gene whose expression is strongly induced by neuronal activity. It regulates synaptic plasticity and learning and memory [27–29] and its dysfunction has been suggested to be involved in autism, bipolar disorder and cognitive disorders [30–32]. Interestingly, Npas4 expression in the hippocampus is increased by excitotoxic and ischemic insults [27, 33, 34]. A growing body of evidence indicates that Npas4 has neuroprotective effects in cell culture and in vivo models of trophic factor deprivation, excitotoxicity, potassium cyanide neurotoxicity, ischemia, epilepsy and neuroinflammation [35–38]. Bdnf is a member of the neurotrophic family of neurotrophic factors [39]. Besides playing a key role in brain development and synaptic activity, Bdnf also promotes neuronal health and activity is believed to contribute to the pathogenesis of several neurodegenerative diseases [39–41]. The Bdnf gene has a complex structure with at least eight non-coding 5′ exons which are spliced to one 3′ coding exon. These transcripts are controlled by distinct promotors and differentially regulated by several cis-acting elements [42].

We describe validating the results of ChIP-Seq for Npas4 and Bdnf using standard ChIP assays. Furthermore, we show that forced expression of HDAC3 inhibits expression of Npas4 and Bdnf mRNAs, consistent with these genes being targets of HDAC3. Our results raise the possibility that the neurotoxic effect of HDAC3 is partly mediated through the reduction of the survival-promoting action of Npas4 and Bdnf resulting from their transcriptional repression.

## Methods

### Materials

All cell culture media and reagents were purchased from Invitrogen and chemicals and reagents were from Sigma-Aldrich, unless stated otherwise. Poly-l-lysine from Trevigen was used to coat the plates for primary neuronal cultures. RGFP966, HDAC3 inhibitor (Sigma-Aldrich, catalog #SML1652) was dissolved in DMSO and used at final concentration of 10 μg/ml. The antibodies used for western blotting are as follows, Bdnf (Aviva Systems Biology, catalog #ARP41970), ERK1/2 (Cell Signaling Technology, catalog #9102), Npas4 (Kempbio, clone #35-4) and HRP-conjugated secondary antibodies (Pierce). For ChIP analysis, Hdac3 antibody (Santa Cruz, catalog #sc-376957) was used. The primary antibodies were used at a dilution of 1:1000 and secondary antibodies were used at 1:20,000 dilution.

### Primary neuronal culture and treatments

Wistar rats were used for all experiments. Founder rats were purchased from Charles River (Wilmington, MA) and housed to generate a colony within the institutional vivarium, which is run by dedicated staff and monitored by a veterinarian. The rats were maintained at 69 °F with a light–dark cycle of 12 h day and 12 h night, with 18% protein rodent pellets (Envigo Teklad Global 2018) and water available ad libitum. For culturing of cerebellar granule cultures (CGNs), 7–8 day old pups were used. The pups were euthanized by carbon dioxide inhalation followed by rapid decapitation. For cortical neurons, embryos were extracted at gestation day 17. The pregnant dam was euthanized by carbon dioxide inhalation followed by rapid decapitation. After extraction, the embryos were euthanized by rapid decapitation [43].

CGNs, prepared from the cerebella of pups, were treated with either 25 mM KCl (high potassium, HK) or without KCl (low potassium, LK) for 6 h as described previously [43]. Briefly, cultures were grown in BME (Invitrogen) supplemented with 10% FBS (Invitrogen). About 20 h after plating, the cultures were treated with 1 μM of Ara-C to prevent mitotic cell proliferation. Both male and female rat pups were used for culturing neurons. For cortical neurons, the cortices of E17 embryos were dissociated with trypsin and the cells were plated in Neurobasal media with B27 supplements). Both male and female rat pups were used for culturing neurons. For pharmacological inhibition of HDAC3, 10 μM of RGFP966 (Sigma) was added to the culture media.

### Adenovirus generation and infection

The HDAC3 adenovirus was generated using ViraPower Adenovirus Expression Kit (Invitrogen) as previously described [44]. Briefly, crude viral lysate was purified by CsCl ultracentrifugation and the titer of the adenovirus was in the range of 10^10^–10^11^ pfu/ml. For infecting neurons with adenovirus, the conditioned media was collected and saved and the neurons were incubated with fresh media sufficient to cover the cell layer along with the virus. The plates were gently swirled every 15 min for 2 h after which the viral media was removed and the conditioned media was returned to the neurons [44]. The gene and protein expression analysis was performed after 28 h.

### RNA preparation and gene expression analysis

Total RNAs were extracted using Trizol reagent according to the manufacturer’s guidelines. For reverse transcription, 1 µg of total RNA was used, and cDNA was prepared using Verso cDNA synthesis kit (Thermo Scientific). Resulting cDNA was used as a template for PCR or qPCR analysis. Quantification of gene expression by RT-qPCR was performed in the Bio-Rad Cycler using the iQ SYBR Green Supermix (Bio-Rad, catalog #1708882). The primers used for qPCR are shown in Table 1 and the PCR amplification was performed as follows, initial denaturation 95 °C for 2 min followed by denaturation at 95 °C for 10 s, annealing at 57 °C for 15 s and extension at 72 °C for 30 s for 40 cycles. Melt curve analysis was performed to verify the amplification of single PCR product. Actin was used as a normalization control and the relative expression levels of transcripts were calculated by the 2^−ΔΔCT^ method.

**Table 1:** Primer pairs used for ChIP analysis and qPCR analysis

ChIP analysis		
Gene	Forward	Reverse
Npas4 ChIP	5′ AGCCCCTTCTCATCCTTTGC	5′ CTTCCTTGCTTCCCGGTCTT
Bdnf ChIP	5′ CACGGCTCTTCCTAGCACTT	5′ TGTTGATGAGACCCTTTCCATGT
cFos ChIP	5′ AGTCTCATCCCCTGACCCTG	5′ CCTCAGCTGGCCGCTTTAT
Foxp1 ChIP	5′ CTTGGAGGATGTTGCTCTGC	5′ GAGAGGAGGATTTCCAGAAGTT
Stat3 ChIP	5′ GTAAGAGGCTCACGGTCTCG	5′ AACCGCTGAATTACAGCCCC

qPCR analysis		
Transcript	Forward	Reverse
Bdnf exon I	5′- CAGGGCAGTTGGACAGTCAT	5′ TACGCAAACGCCCTCATTCT
Bdnf exon II	5′ TTCGGCTCACACTGAGATCG	5′ CAGTATACCAACCCGGAGCTT
Bdnf exon III	5′ TTGGAGGGCTCCTGCTTTCT	5′ CTGGGCTCAATGAAGCATCCAG
Bdnf exon IV	5′ ACTGAAGGCGTGCGAGTATT	5′ TGGTGGCCGATATGTACTCC
Bdnf exon V	5′ AAACCATAACCCCGCACACT	5′ CTTCCCGCACCACAGAGCTA
Bdnf exon VI	5′ GATGAGACCGGGTTCCCTCA	5′ TTGTTGTCACGCTCCTGGTC
Bdnf exon VII	5′ ACTGTCACCTGCTTTCTAGGG	5′ GAGTTCCGCAGACCCTTTCA
Bdnf exon VIII	5′ GTGCTCAGGCTAATCCTCGTT	5′ CTTTCTCCTGGGATGCACAGT
Bdnf exon IXA	5′ ACGGCGTGAACAGAGATCAT	5′ ACGGTTTCTAAGCAAGTGACG
Npas4	5′ CAGATCAACGCCGAGATTCG	5′ GACACCCTTGCGAGTGTAGAT
Actin	5′ GCCATGTACGTAGCCATCCA	5′ GGAGCGCGTAACCCTCATAG

Table lists the primers used for ChIP and qPCR analysis. The exon specific Bdnf primers described in Nair et al. [83] were used for the analysis

### Chromatin immunoprecipitation (ChIP): 7–8 days after plating

CGNs were subjected to serum-free HK or LK media as described previously. ChIP was performed as described previously [45]. Briefly, after treatment with HK or LK medium, the neurons were fixed in 1% formaldehyde for 10 min at room temperature. Fixation was stopped by adding glycine (0.125 M). After washing twice in ice cold PBS, cells were scraped in PBS and centrifuged at 1500 rpm for 10 min. Pellets were suspended in 800 µl of buffer 1 (50 mM Hepes–KOH, pH 7.5; 140 mM Nacl; 1 mM EDTA pH 8; 10% Glycerol; 0.5% NP-40; 0.25% Triton X-100; supplemented with 1 mM PMSF; and Protease inhibitor cocktail). Cells were lysed for 10 min on ice and nuclei were pelleted by centrifugation at 3000 RPM for 10 min. After washing the nuclei once with buffer 2 (200 mM Nacl; 1 mM EDTA pH 8; 0.5 mM EGTA, pH 8; 10 mM Tris Hcl, pH 8 supplemented with 1 mM PMSF; and Protease inhibitor cocktail), sonication was performed in buffer 3 (1 mM EDTA, pH 8; 0.5 mM EGTA, pH 8; 10 mM Tris HCl, pH 8.1; 0.5% SDS supplemented with 1 mM PMSF and Protease inhibitor cocktail). Chromatin was sonicated to a size range of 100–300 bp fragments for ChIP-Seq and 800–1000 bp fragments for normal ChIP analysis. Samples were centrifuged at 13,000 rpm for 15 min at 4 °C. One hundred micrograms of chromatin was used for each ChIP analysis. Samples were incubated overnight with 3–5 µl of antibody (1 µg/µl). Precipitation and washing of chromatin samples has been described earlier. Finally, DNA was suspended in 60 µl of 10 mM Tris, pH 7.5. For PCR, 3 µl of template DNA was used for amplification. The promoter primer sequences used are provided in Table 1.

### ChIP-Seq and data analysis

For sequencing, ChIP was performed as described earlier and immunoprecipitated DNA was used for the library construction using NEB Next kit (New England Bio Labs). Library prepped DNA samples were sequenced on an Illumina HiSeq 2000 at the University of Southwestern Medical Center sequencing facility using default parameters (single end, forward sequencing). Quality assessment of the raw sequencing reads was done using NGS-QC-Toolkit. Sequencing reads with quality score under Phred Score < 20 were discarded. The quality filtered reads were then aligned to rat reference genome RGSC_v3.4 (rn4) using Bowtie2 (v 2.0.6) aligner. Transcription factor binding sites were identified using the peak calling algorithm QuEST [46]. QuEST employs a kernel density estimation approach to determine positions where protein complexes contact DNA. Peaks were called for regions with fold change values greater than three and q-value obtained by Bonferroni correction of p-values greater than 0.00001. Once peak regions were called, the HOMER pipeline was used to annotate the peaks. Direct quantitative comparison in HK and LK samples was done using MAnorm. Based on the assumption that most common peaks should be the same between two ChIP-Seq samples, MAnorm first normalizes all the peaks based on a robust regression model and then identifies those peaks which are statistically significantly expressed in one sample compared to the other.

### Luciferase assay

For generating the Npas4-Luciferase construct, the genomic fragment from − 2 kb to + 100 b of Npas4 gene was amplified using primers and cloned into PGL3-basic vector (Promega) using the XhoI/HindIII restriction sites. For the Bdnf-Luciferase construct, a 500 b genomic fragment was amplified and cloned using the ChIP primers mentioned in Table 1. For checking the promoter activity, HT22 cells were transfected with 4 μg of either HDAC3 or GFP plasmids, along with 4 μg of the promoter plasmid in PGL3-firefly luciferase and 0.4 μg of plasmid of renilla luciferase plasmid to normalizing the transfection efficiency. The luciferase activity was measured using the Promega Dual Luciferase Reporter System and the relative luminescence units (RLU) obtained with firefly luciferase were normalized with the RLU of renilla luciferase.

### Statistical analysis

The data analysis and generation of graphs were done using GraphPad Prism 5 software. The intensity of bands was quantified using ImageJ. Statistical analysis was performed using two-tailed Student t-test for comparing two groups and for multiple group comparisons, ANOVA analysis was performed. The significance levels are indicated based on the p-value as follows, *< 0.05, **< 0.01, ***< 0.001. The results are displayed as mean ± SD.

## Results

### Genome wide mapping of Hdac3 binding

**Fig. 1:**
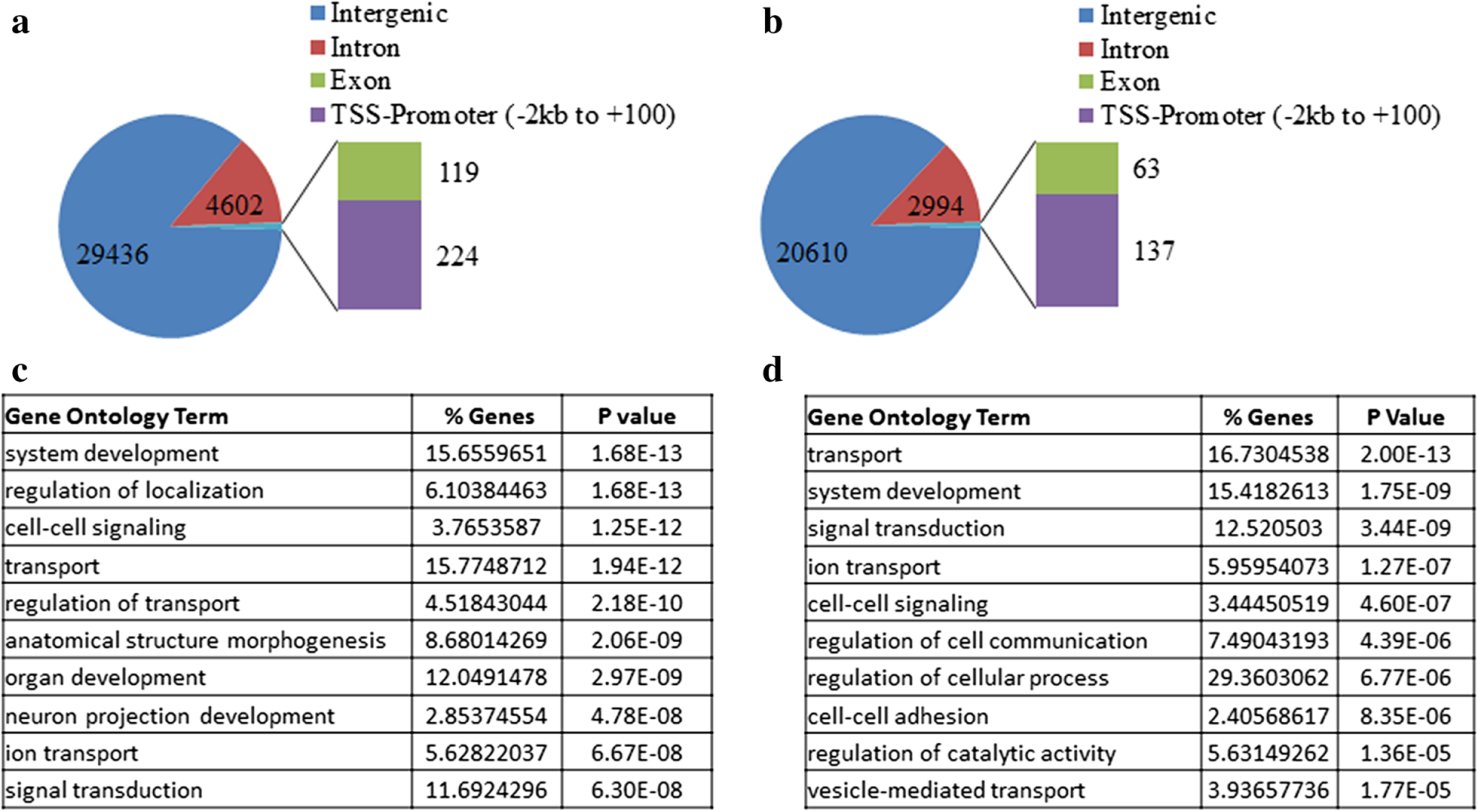
Genome-wide characterization of HDAC3 peak distributions and GO term enrichments. Pie charts in HK (**a**) and LK (**b**) show distribution of HDAC3 binding sites in different area of genome. Maximum number of peaks were identified in intergenic regions. **c**, **d** Biological processes GO term analysis was performed in the groups of genes that show increased HDAC3 binding at the promoter region (− 2 kb to + 100 b of TSS. GO result from Database for Annotation, Visualization and Integrated Discovery (DAVID)

Cultured CGNs undergo apoptosis when switched from medium containing depolarizing levels of potassium (HK medium) to non-depolarizing medium (LK) [47]. Using this widely utilized model of neuronal apoptosis we showed that forced expression of HDAC3 induces death of otherwise healthy neurons (treated with HK medium) and greatly exacerbated the extent of death in LK. As a step towards identification of transcriptional targets of HDAC3 that regulate its neurotoxic activity, we used three independent sets of HK and LK treated CGNs. RNA from sister cultures were subjected to RT-PCR analysis to confirm that the expression of genes known to be altered in this paradigm, such as the upregulation of c-jun, were indeed altered (data not shown). After this “quality control” step, the cultures were processed for ChIP-Seq analysis. To correct genomic copy number variations and sonication introduced fragment bias, input control (chromatin taken before immunoprecipitation) was used. Table 2 shows the details of the mapping statistics. Using criteria of fold change greater than three and FDR less than 0.00001 a total number of 34,450 and 23,850 genomic binding sites for HDAC3 were revealed in HK and LK respectively. Binding sites were grouped depending on their position relative to the nearest gene. Figure 1a, b shows the distribution of these sites in the whole genome. Overall, HDAC3 was preferentially associated with intergenic and intronic regions. Others have also shown maximum enrichment of transcription factors and co-regulators including HDAC3 in intergenic and intronic regions [48, 49]. To identify transcriptional targets of the HDAC3, we focused on around 2 kb upstream TSS region of the nearest gene, considered as promoter, and found 224 and 137 high occupancy sites in HK and LK respectively (Fig. 1a, b). Table 3 shows some of the neuronal genes with HDAC3 binding in the promoter region. A detail list of all the binding sites is listed in Additional file 1: Table S1 and Additional file 2: Table S2. Next, we analyzed important functional groups of genes that show increased HDAC3 binding at their promoter region and did the gene ontology (GO) terms enrichments. Top GO analysis for the genes promoter bound with HDAC3 include enrichment of GO terms specific for neuronal functions. These include neuron projection development, system development, vesicle mediated transport, ion transport, signal transduction, and regulation of cell communication etc. (Fig. 1c, d). Details of all the GO terms are given as a separate excel sheet in Additional file 3: Table S3A, B.

**Table 2:** ChIP-Seq read analysis

Sample	Raw read count	Unique read count	Mapped read count	Mapping rate (%)
HK	28,649,979	11,631,206	8,640,236	74
HK_input	25,431,774	20,338,856	15,224,517	75
LK_H3	29,817,952	12,785,551	9,500,783	74
LK_input	14,549,174	11,003,800	5,934,983	54

Table contains the number and percentage of high quality uniquely mapped reads. Reads that were assigned uniquely to the rat genome were used in the analysis

**Table 3:** Genes with HDAC3 binding sites in cerebellar granule neurons

Gene symbol	Gene details	Distance to nearest TSS	Binding in HK/LK
Nr3c2	Nuclear receptor subfamily 3, group C, member 2	− 2023	HK
Npy2r	Neuropeptide Y receptor Y2	− 1951	HK
Opa3	Optic atrophy 3	− 1853	HK
Neurod2	Neuronal differentiation 2	− 1583	HK
Hes1	hes family bHLH transcription factor 1	− 1363	LK
Npas4	Neuronal PAS domain protein 4	− 1063	HK
Bcl2l10	BCL2-like 10 (apoptosis facilitator)	− 1051	LK
Bdnf	Brain-derived neurotrophic factor	− 1015	LK
Hrk	Harakiri, BCL2 interacting protein	− 917	LK
Fadd	Fas (TNFRSF6)-associated via death domain	− 900	HK
Nptxr	Neuronal pentraxin receptor	− 890	HK
C1qa	Complement component 1, q subcomponent, A chain	− 874	HK
Nmnat2	Nicotinamide nucleotide adenylyltransferase 2	− 868	HK
Bid	BH3 interacting domain death agonist	− 824	LK
Rhebl1	Ras homolog enriched in brain like 1	− 687	LK
Grip1	Glutamate receptor interacting protein 1	− 375	LK
Klf10	Kruppel-like factor 10	− 362	HK
Diablo	Diablo, IAP-binding mitochondrial protein	− 284	LK
Grm4	Glutamate receptor, metabotropic 4	− 259	HK
Npffr1	Neuropeptide FF receptor 1	− 202	LK
Tgfb1	Transforming growth factor, beta 1	− 112	LK
Syngr1	Synaptogyrin 1	− 87	LK
Plxnd1	Plexin D1	− 45	HK
Gabarap	GABA(A) receptor-associated protein	− 31	HK
App	Amyloid beta (A4) precursor protein	52	LK
Snapin	SNAP-associated protein	58	HK

Table lists some of the neuronal genes with HDAC3 binding site in the promoter region. The genes are sorted based on the distance of the binding site from the TSS

### Promoter occupancy of Hdac3 and gene expression analysis

**Fig. 2:**
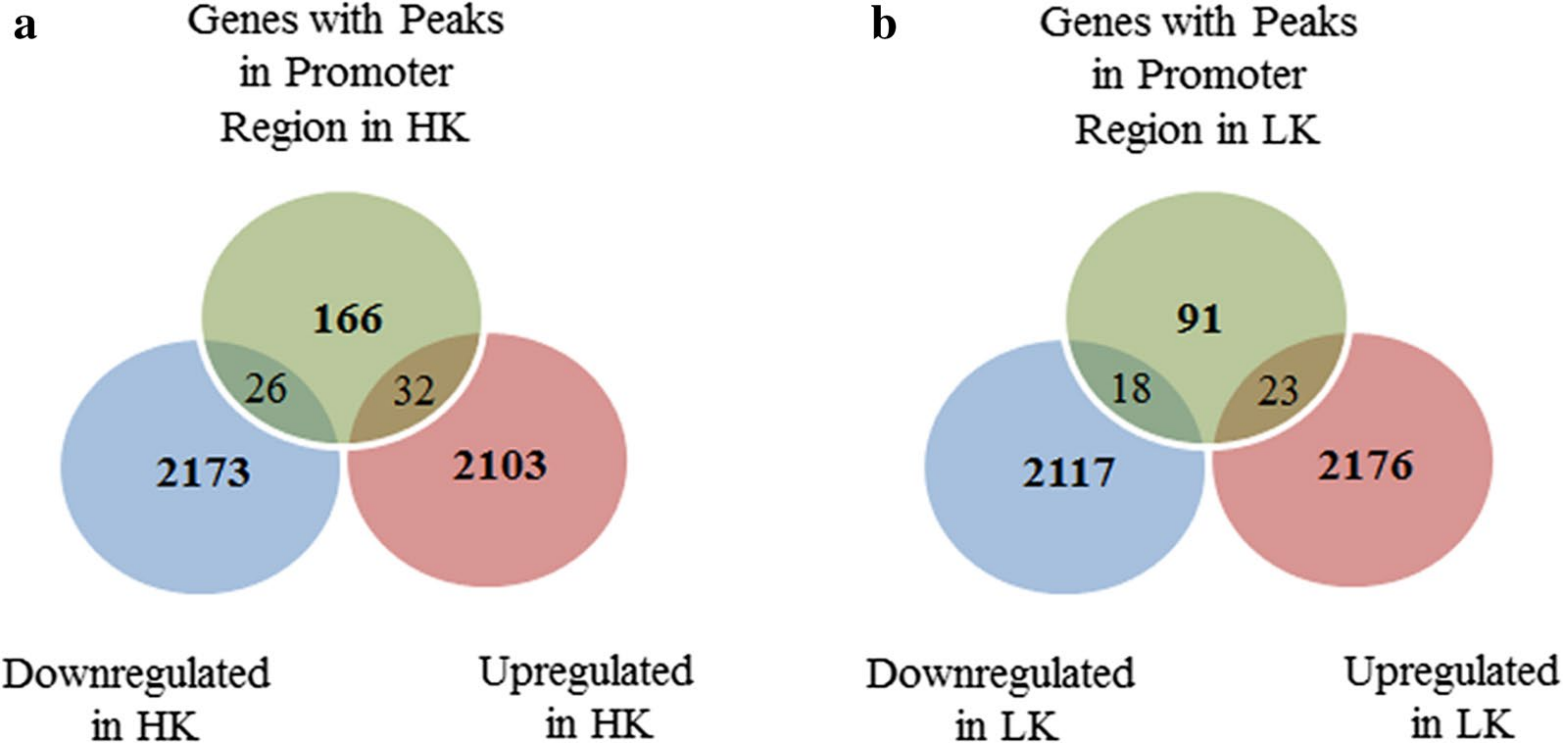
Integrative analysis of ChIP-Seq and RNA-Seq data [43] identifies multiple HDAC3 targets in HK and LK. Venn diagram shows HDAC3 occupancy at the promoter region of differentially-expressed genes in either HK (**a**) or LK (**b**) condition. List of differentially-expressed genes during neuronal death [43] was used and overlapping was performed between the number of genes up or down regulated having HDAC3 occupancy at the promoter

We previously reported the results of a RNA-Seq study identifying differentially expressed genes in CGNs treated with HK and LK [43]. We used that dataset and compared the promoter binding data of HDAC3 from the current study to identify the overlapping genes. The results of this comparison are shown in Fig. 2a, b. Overall under HK condition, out of 224 genes that were showing HDAC3 promoter occupancy, only 58 genes showed differential expression (26 down and 32 up regulated). Similarly, in LK condition, from a list of 137 genes, only 41 showed differential expression (18 down and 23 up regulated). An Excel spreadsheet containing this information is provided (Additional file 4: Table S4). It is likely that genes that overlap are regulated by HDAC3 or that their expression change is the consequence of HDAC3 action on direct targets.

### Validation of ChIP Seq analysis

**Fig. 3:**
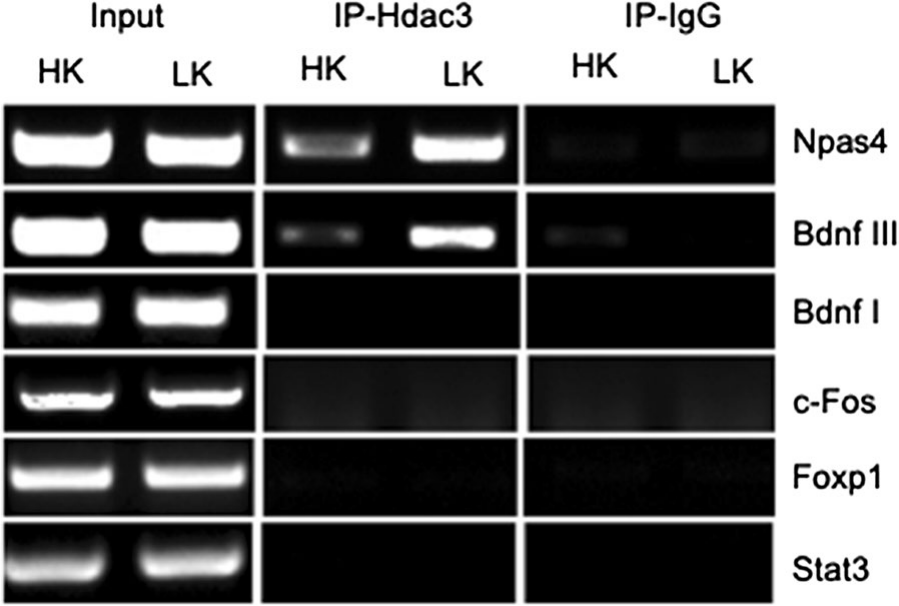
PCR validation of ChIP-Seq data. ChIP was used to validate the ChIP-Seq results. For this, ChIP was conducted on genes that were positive for HDAC3 occupancy based on ChIP-Seq (Npas4 and Bdnf) and three genes known to have neuroprotective effects but were not among the positive hits in the ChIP-Seq (cFos, FoxP1, Stat3). PCR was conducted following immunoprecipitation of sheared chromatin using primers to the promoter regions of these genes (see Table 1 for primer sequences)

To validate the ChIP-Seq data we selected two genes showing positive binding in the promoter region, Npas4 and Bdnf, and conducted standard ChIP-PCR assays. These genes were analyzed along with three other genes that are also known to have neuroprotective effects but were not among the genes identified in the ChIP Seq analysis—the neuronal activity-depended gene c-Fos, the neuroprotective Forkhead protein FoxP1, and the Stat3 transcription factor. ChIP was conducted using CGN cultures treated with HK or LK medium and focusing on the region spanning about 1000 bp upstream of the transcription-start site region. HDAC3 occupancy was detectable at the promoter region of the Npas4 and Bdnf genes. Both genes showed HDAC3 occupancy in HK and the enrichment robustly increased in LK (Fig. 3). In contrast, association of HDAC3 with the promoters of the other three genes, cFos, FoxP1 and Stat3 was not detectable either in HK or LK. Bdnf genomic structure contains multiple promoters, used to generate different transcripts [42]. The best characterized Bdnf promoter is immediately upstream of the rat exon III, which corresponds to the mouse Bdnf exon IV promoter [50]. For our studies this Bdnf III promoter, cloned from rat genomic DNA, was used since it is shown to be activated upon membrane depolarization in cultured cortical and hippocampal neurons by means of KCl (50 mM) treatment [51–54]. We also analyzed Bdnf I promoter and found out that the HDAC3 enrichment was specific to the Bdnf III promoter as there was no binding seen on the Bdnf I promoter (Fig. 3). Although limited in scope, these findings indicate that the ChIP-Seq analyses accurately identified genes that were bound by HDAC3.

### Npas4 and Bdnf expression is negatively regulated by HDAC3 in neurons

**Fig. 4:**
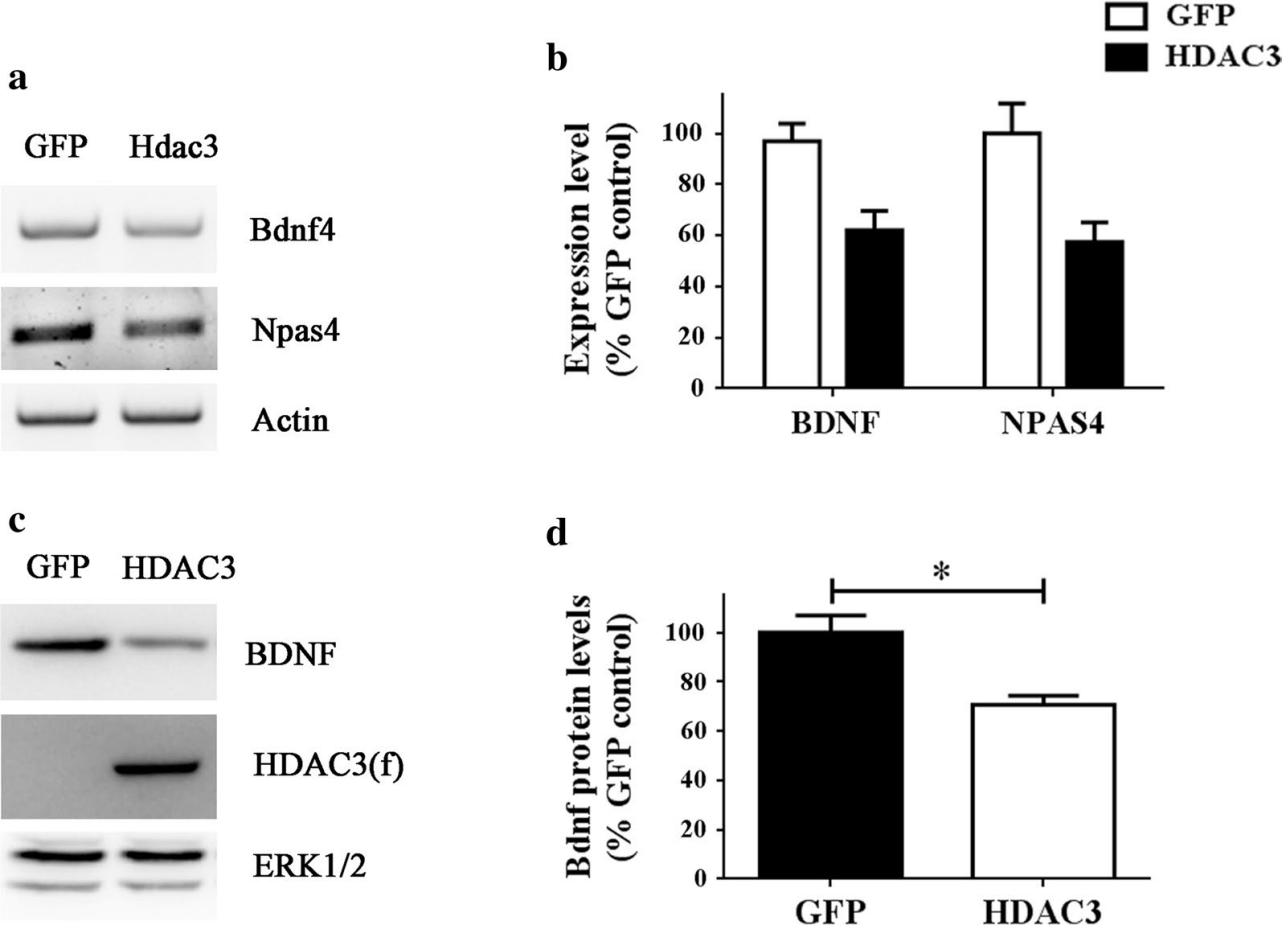
Overexpression of HDAC3 causes suppression of Npas4 and Bdnf. **a**, **b** RT-PCR analysis mRNA isolated from cortical neurons that were transduced with adenovirus expressing GFP or HDAC3 (n = 3). **c**, **d** Western blot analysis of Bdnf protein levels in cortical neurons transduced with adenovirus expressing GFP or HDAC3 (n = 3)

To examine if association of HDAC3 with the Npas4 and Bdnf gene promoters resulted in altered expression, we overexpressed HDAC3 in cortical neurons using adenovirus and evaluated its effect on Npas4 and Bdnf gene expression. Since extended overexpression of HDAC3 kills cortical neurons [15], we limited expression to 28 h. Both Bdnf and Npas4 mRNA levels were downregulated by HDAC3 (Fig. 4a, b). The downregulation of Bdnf was also detected at the protein level (Fig. 4c, d). Due to the very low expression of Npas4 under basal conditions we could not detect the reduction of Npas4 protein.

**Fig. 5:**
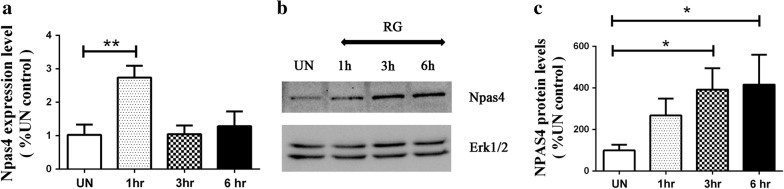
Inhibition of HDAC3 by RGFP966 stimulates Npas4 expression. **a** Cortical neurons were treated with 10 μM RGFP966 and the expression levels of Npas4 was determined at different time points by qPCR (n = 3) and the protein levels were also analyzed by western blotting (**b**, **c**) (n = 3)

**Fig. 6:**
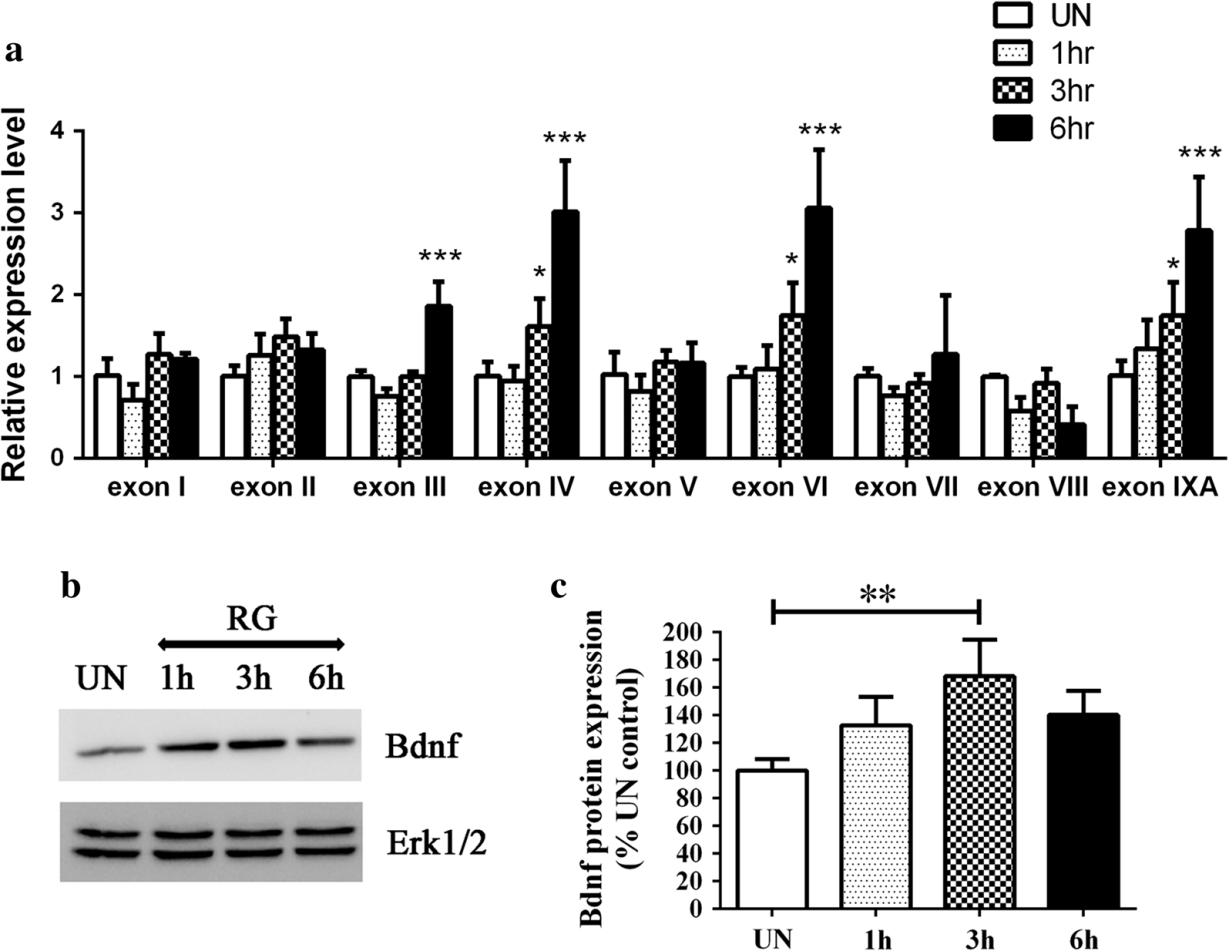
Inhibition of HDAC3 by RGFP966 causes upregulation of specific Bdnf exons. **a** Cortical neurons treated with RGFP966 were analyzed with exon specific primers for Bdnf [83] and a significant upregulation was observed in exons III, IV, VI and IXA (n = 3). **b**, **c** The protein levels were analyzed by western blotting which shows an increase in Bdnf expression (n = 3)

To confirm that Npas4 and Bdnf are targets of HDAC3 we treated cortical neurons with RGFP966, a chemical inhibitor of HDAC3. It is well established that RGFP inhibits HDAC3 highly selectively and effectively both in cell culture and in vivo [17, 20, 22]. We found an upregulation of Npas4 (Fig. 5) and Bdnf (Fig. 6) expression at the mRNA and protein level following treatment of neurons with RGFP966. We also analyzed various transcripts of Bdnf using exon specific primers and found that the transcripts corresponding to exons III, IV, VI and IXA of the Bdnf gene were the most induced by HDAC3 inhibition.

**Fig. 7:**
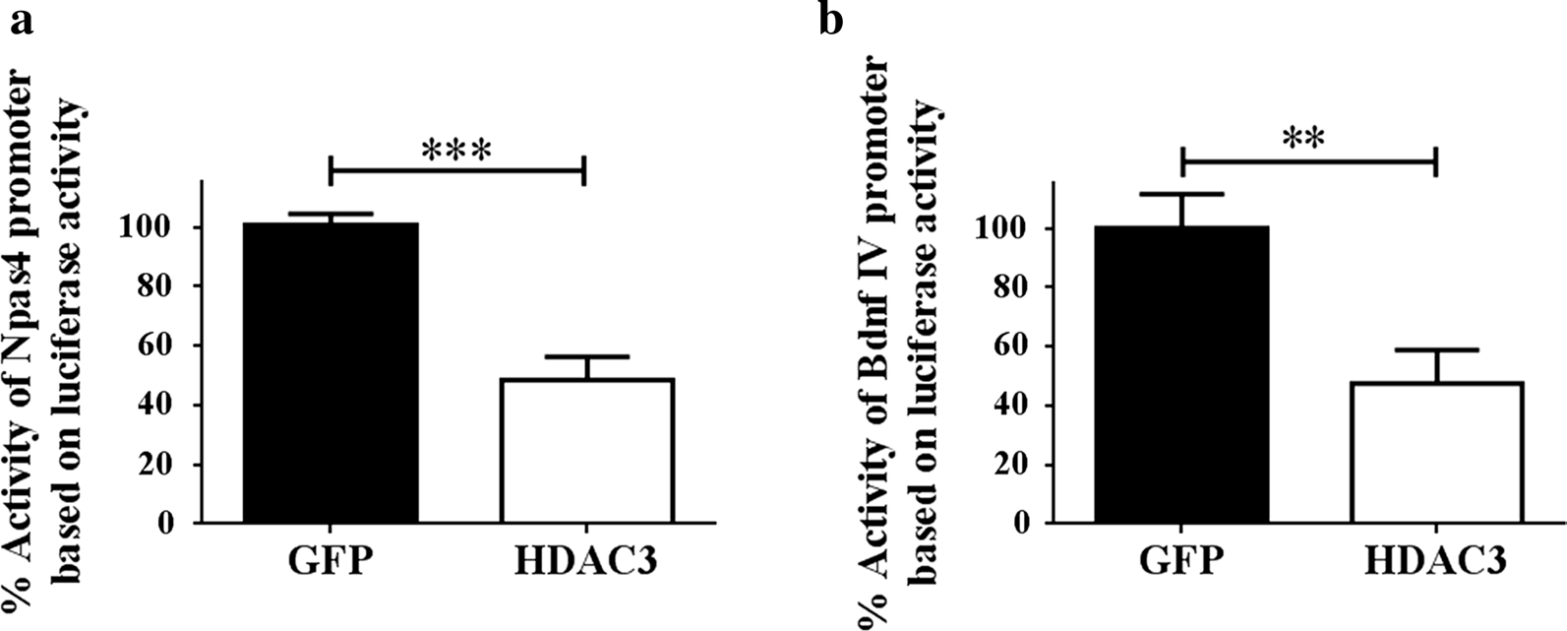
HT22 cells were transfected with either GFP or HDAC3 plasmids along with renilla luciferase and PGL3-basic vector with the promoter sequence of **a** Npas4 or **b** Bdnf. The RLU values of firefly luciferase obtained were normalized with the RLU of renilla luciferase and the promoter activity is expressed as percentage of the activity observed in GFP transfected control (n = 3)

Lastly, we performed transcriptional activity assays using the Npas4 and Bdnf III promoters fused to luciferase reporter in the pGL3-basic vector. As shown in Fig. 7, co-expression of HDAC3 reduced the activity of both the Npas4 and Bdnf III promoters. Taken together, the results of our ChIP assays, expression analyses, pharmacological treatment and promoter activity analysis indicate that the Npas4 and Bdnf genes are transcriptionally repressed by HDAC3.

## Discussion

Forced expression of HDAC3 promotes death of neuronal cells, but not of non-neuronal cell types [15] suggesting unique targets of HDAC3 in neurons that are regulated during neuronal death. Transcriptional targets of HDAC3 have been described in non-neuronal systems [23–25]. However, considering the role of HDAC3 in brain, its targets in healthy and apoptotic neurons have not been previously described. Our ChIP-Seq analysis has identified several potential HDAC3 targets of which we have followed up on two, Npas4 and Bdnf. Expression of both these genes is stimulated by neuronal activity. More importantly, both genes have been shown to have neuroprotective actions. This is particularly true for Bdnf for which neuroprotective effects have been amply demonstrated in a variety of cell culture and in vivo models of neurodegenerative disease [39–41].

It is important to mention here that in our ChIP-Seq analysis, we could not detect HDAC3 peak at the NPAS4 promoter in LK, however, repeated ChIP-PCR validation data clearly showed that HDAC3 was not only present at the promoter in LK condition, its binding was highly increased. At present, we don’t have clear explanation for this discrepancy, however, it is possible that under LK condition, neurons are primed to undergo apoptosis that may lead to increased background noise resulting in the loss of important information.

Although one of several IEGs induced by neuronal activity, Npas4 is different from the other activity-induced genes in that it is expressed specifically in neurons and its expression is not increased by other extracellular stimuli, such as growth and neurotophic factors [55, 56]. The mechanisms regulating transcription of several IEGs including Npas4 in neurons reveal that the machinery required for transcription initiation is in place at the promoter even in the absence of neuronal activity but RNA polymerase stalls just downstream of the transcription start site [57]. A recent report revealed that in the absence of neuronal activity the conformation of chromatin stearically hinders the activation of transcription of these genes [58]. Double-strand breaks formation within the upstream regions following stimulation of neuronal activity relieves this hindrance permitting pre-assembled transcriptional machinery to transcribe these genes [58]. We propose that another factor contributing to suppression of Npas4 gene transcription is HDAC3. Interestingly, HDAC3 does not associate appreciably with the promoter region of the c-Fos gene suggesting that neuronal activity stimulates the Npas4 and c-Fos genes by different mechanisms.

Results of other studies indicate that the Bdnf gene is a target of Npas4 [27, 59, 60]. Indeed, Npas4 has been found to associate with the mouse Bdnf-I and Bdnf-IV (similar to rat Bdnf III) promoters, which are also activated by neuronal activity [27]. This explains the delayed induction of Bdnf expression in comparison to the increase in Npas4 transcription following neuronal excitation [27]. We find the Bdnf III promoter, but not the Bdnf-I promoter, is bound by HDAC3 suggesting that the expression of these two Bdnf transcripts may also be subject to different regulatory mechanisms. Interestingly, this Bdnf promoter is selectively downregulated in Huntington’s disease and other neurodegenerative conditions [61]. Our finding that HDAC3 downregulates Bdnf expression appears to contradict a recent report which described that HDAC3 is recruited to the Bdnf promoter by MeCP2 [62]. However, the authors of that study found that Bdnf is reduced in HDAC3 conditional knock out mice suggesting that HDAC3 positively regulates Bdnf gene transcription [62]. There have also been studies where Bdnf was found to be repressed by HDAC3 [63–65]. It is possible that whether HDAC3 suppresses or stimulates Bdnf expression may depend on the transcriptional regulators it associates with at the Bdnf promoter. We previously described that in HD-related neurodegeneration HDAC3 disassociates with normal huntingtin and associates with HDAC1 [13, 14]. Moreover, HDAC3 requires phosphorylation by GSK3β to become neurotoxic [15].

Association of HDAC3 with the Npas4 and Bdnf gene promoters is robustly elevated in CGNs treated with LK. This may explain the reduction of expression of the Npas4 and Bdnf mRNAs in neurons primed to die. Given that both genes have protective effects in many different models [34, 37, 66], the inhibition of their expression by HDAC3 might explain its neurotoxic effect. Indeed, extracellular Bdnf has been shown to protect CGNs from LK-induce death [67–71].

In addition to their roles in neuroprotection, both Npas4 and Bdnf enhance learning and memory and cognitive function [29, 72–74]. Interestingly, elevating HDAC3 expression negatively regulates learning and memory whereas ablation of the HDAC3 gene or pharmacological inhibition of HDAC3 restores it [75–78]. It is possible that the negative effect of HDAC3 on learning and memory is also mediated by the transcriptional repression of Npas4 and Bdnf.

## Conclusion

We provide evidence indicating that Npas4 and Bdnf, two genes that have critical roles in brain development and function, are transcriptional targets of HDAC3-mediated repression. HDAC3 inhibitors have been shown to protect against behavioral deficits and neuronal loss in mouse models of neurodegeneration [7, 17, 79–81]. It is possible that these effects involve upregulation of Npas4 and Bdnf, both of which have been described to be neuroprotective. We recently described that genetic ablation of HDAC3 itself affects brain development [82]. Further work is needed to determine whether these effects on brain development involve deregulation of Npas4 and Bdnf gene expression.

## Supplementary information

**Additional file 1: Table S1.** Unique HDAC3 Peaks in the promoter region in HK. Table shows a list of putative transcriptional targets of HDAC3 in HK. Genes were grouped based on the enrichment of HDAC3 in their promoter region. An area of around 2kb upstream of the TSS was considered as promoter.

**Additional file 2: Table S2.** Unique HDAC3 Peaks in the promoter region in LK. Table shows a list of putative transcriptional targets of HDAC3 in LK. Genes were grouped based on the enrichment of HDAC3 in their promoter region. An area of around 2kb upstream of the TSS was considered as promoter.

**Additional file 3: Table S3.** Biological Process-Gene Ontology. List of putative transcriptional targets of HDAC3 in HK (A) and in LK (S3B) were subjected to DAVID and functional annotation was performed. p value and benjamini FDR were calculated and the genes were categorized based on their cellular function.

**Additional file 4: Table S4.** DEGs having HDAC3 peaks in promoter region. Previously published data [77] of differentially-expressed genes during neuronal death was utilized and overlapping analysis with the ChIP-Seq data was performed to get the expression of genes having HDAC3 occupancy at the promoter. S4A shows genes in HK, and S4B shows genes having differential expression in LK.

## Data Availability

All reagents generated as part of this study are available on request. All data is published in the manuscript and additional results.

## Abbreviations

HDAC3: histone deacetylase-3
Bdnf: brain-derived neurotrophic factor
Npas4: neuronal PAS domain protein 4
ChIP-Seq: chromatin immunoprecipitation-sequencing
HK: high potassium
LK: low potassium
CGNs: cerebellar granule neurons
